# Functional Outcome in Limb-Salvage Surgery for Soft Tissue Tumours of the Foot and Ankle

**DOI:** 10.1080/13577149778326

**Published:** 1997-06

**Authors:** Nigel R. Colterjohn, Aileen M. Davis, Brian O'Sullivan, Charles N. Catton, Jay S. Wunder, Robert S. Bell

**Affiliations:** 1 University Musculoskeletal Oncology Unit and Division of Orthopaedic Surgery Mount Sinai Hospital and the University of Toronto Canada; 2 Department of Radiation Oncology Princess Margaret Hospital and the University of Toronto Canada; 3 Suite 476 Mount Sinai Hospital 600 University Avenue Toronto Ontario M5G 1X5 Canada

## Abstract

*Purpose*. This paper describes the functional and oncologic outcome of 30 cases (in 29 patients) treated with limb-salvage surgery for localized soft tissue sarcoma (STS) or fibromatosis of the foot and ankle.

*Subjects*. Patients were eligible for the study if they had a STS or fibromatosis in the distal one-third of the tibia or the foot such that ablative surgery would require a below-knee amputation; had no metastatic disease at presentation; and had a minimum of 2 years of follow-up.

*Methods*. Function was prospectively evaluated using the modified Enneking functional rating scale (MSTS) at 3, 6, 12 months and at most recent follow-up. Premorbid work status and change following surgery, lower leg oedema, and the use of orthotics and ambulatory aids were consecutively assessed. Tumour characteristics were recorded and patients were followed for systemic and local recurrence.

*Results*. Thirty-six consecutive cases were managed by a multi-disciplinary sarcoma team. Six patients underwent below-knee amputation due to extensive local disease, while 30 cases were treated with limb-salvage surgery. Of the patients treated with limb salvage, there were 19 high-grade sarcomas, five low-grade sarcomas and six cases of fibromatosis. Microscopically negative margins were achieved in 26 of 30 cases. Ten cases required bone excision, and eight patients needed free vascularized tissue flaps. Twenty-five patients received adjuvant radiotherapy. Seven patients had post-operative complications. At mean follow-up of 52 months (range 24–109 months), four patients had developed systemic recurrence. There was one local recurrence in a patient with fibromatosis, while another patient with fibromatosis developed recurrence a considerable distance from the surgical and radiation field. Mean scores on the MSTS were 27.5 (range 11–35), 29.9 (range 13–35), 31.4 (range 17–35) and 31.0 (range 13–35) at 3, 6, 12 months and at most recent follow-up, respectively. Eighty-five per cent of the patients scored good to excellent at their last visit. Twelve patients reported persistent pain with two continuing to require occasional narcotics. Six had persistent mild oedema. Four required shoe modifications and three continue to use a cane. Six patients were unable to return to their premorbid employment with the majority of these previously employed in jobs requiring physical labour or long periods of either standing or walking.

*Discussion*. Thirty of 36 patients (83%) presenting with foot and ankle STS or fibromatosis were candidates for limb preservation. With excellent local control and good functional outcome demonstrated in this study, limb salvage should be a primary goal in the management of selected patients with STS and fibromatosis of the foot and ankle.


*Sarcoma* (1997) 1, 67–74

ORIGINAL ARTICLE

### Functional outcome in limb-salvage surgery for soft tissue tumours of the foot and ankle

NIGEL R. COLTERJOHN,[1] AILEEN M. DAVIS,[1] BRIAN O'SULLIVAN,[2] CHARLES N. CATTON,[2] JAY S. WUNDER[1] & ROBERT S. BELL[1]

[1]*University Musculoskeletal Oncology Unit and Division of Orthopaedic Surgery, Mount Sinai Hospital and the University of Toronto &* [2]*Department of Radiation Oncology, Princess Margaret Hospital and the University of Toronto, Canada*

**Abstract**

*Purpose.* This paper describes the functional and oncologic outcome of 30 cases (in 29 patients) treated with limb-salvage surgery for localized soft tissue sarcoma (STS) or fibromatosis of the foot and ankle.

*Subjects.* Patients were eligible for the study if they had a STS or fibromatosis in the distal one-third of the tibia or the foot such that ablative surgery would require a below-knee amputation; had no metastatic disease at presentation; and had a minimum of 2 years of follow-up.

*Methods.* Function was prospectively evaluated using the modified Enneking functional rating scale (MSTS) at 3, 6, 12 months and at most recent follow-up. Premorbid work status and change following surgery, lower leg oedema, and the use of orthotics and ambulatory aids were consecutively assessed. Tumour characteristics were recorded and patients were followed for systemic and local recurrence.

*Results.* Thirty-six consecutive cases were managed by a multi-disciplinary sarcoma team. Six patients underwent below-knee amputation due to extensive local disease, while 30 cases were treated with limb-salvage surgery. Of the patients treated with limb salvage, there were 19 high-grade sarcomas, five low-grade sarcomas and six cases of fibromatosis. Microscopically negative margins were achieved in 26 of 30 cases. Ten cases required bone excision, and eight patients needed free vascularized tissue flaps. Twenty-five patients received adjuvant radiotherapy. Seven patients had post-operative complications. At mean follow-up of 52 months (range 24–109 months), four patients had developed systemic recurrence. There was one local recurrence in a patient with fibromatosis, while another patient with fibromatosis developed recurrence a considerable distance from the surgical and radiation field. Mean scores on the MSTS were 27.5 (range 11–35), 29.9 (range 13–35), 31.4 (range 17–35) and 31.0 (range 13–35) at 3, 6, 12 months and at most recent follow-up, respectively. Eighty-five per cent of the patients scored good to excellent at their last visit. Twelve patients reported persistent pain with two continuing to require occasional narcotics. Six had persistent mild oedema. Four required shoe modifications and three continue to use a cane. Six patients were unable to return to their premorbid employment with the majority of these previously employed in jobs requiring physical labour or long periods of either standing or walking.

*Discussion.* Thirty of 36 patients (83%) presenting with foot and ankle STS or fibromatosis were candidates for limb preservation. With excellent local control and good functional outcome demonstrated in this study, limb salvage should be a primary goal in the management of selected patients with STS and fibromatosis of the foot and ankle.

**Key words:** *soft tissue sarcoma, fibromatosis, foot, ankle, limb-salvage surgery.*

### Introduction

The treatment of soft tissue sarcoma (STS) in the extremity has changed markedly in the past 30 years. Historically, surgical excision by amputation was the mainstay of management of STS of the extremities. Subsequently, Simon and Enneking showed that achieving local control at the primary site by surgical means alone required radical local resection or amputation.[1] Although improvements in local recurrence rates were observed with radical surgery, the extent of resection often dramatically altered limb function. More recently, advances in radiological imaging and the addition of irradiation as adjuvant therapy following local excision have resulted in acceptable rates of both local recurrence and extremity function.[2–9] Combined management with irradiation and surgery has therefore become the treatment of choice in resectable extremity STS.[2–6,9].

Correspondence to: R. S. Bell, Suite 476, Mount Sinai Hospital, 600 University Avenue, Toronto, Ontario, Canada M5G 1X5. Tel: +1 416 586 8678; Fax: +1 416 586 8397.

1357-714X/97/020067-08 $9.00 ©1997 Carfax Publishing Ltd

Aggressive fibromatosis is a non-encapsulated benign mesenchymal neoplastic process that exhibits local tumour spread and invasion without respect for tissue planes. Both the disease and its treatment can lead to significant functional morbidity. Although fibromatosis does not metastasize, the invasiveness of this tumour is similar to the local behaviour of STS[10–12] and there is a high risk of recurrence following surgical excision alone. The optimal therapy for fibromatosis is controversial and treatment recommendations vary from a radical surgical resection to observation. When eradication of the fibromatosis is warranted due to the functional morbidity of the local disease, combined wide surgical excision and adjuvant radiotherapy offers the best chance of success.[10–12] In this situation, the surgical management parallels that of extremity STS.

The goal of achieving both complete surgical excision of a STS or fibromatosis while maintaining adequate limb function is particularly difficult in the foot and ankle region. Soft tissue tumours of the foot and ankle are rare and the majority of soft tissue lesions in this location are benign, accounting for the high rate of initial simple excision of unsuspected sarcomas and a delay in referral to a sarcoma centre.[13–15] The weight-bearing demands of the distal lower extremity, the limitations of soft tissue coverage and the complexity of underlying vital structures are factors that complicate the planning of limb-salvage surgery. While the literature emphasizes the oncologic outcome in the management of tumours of the foot, there is little information describing the functional and psychological outcome, or the impact of complications, following limb salvage of the foot and ankle.[3,4,6,7,16–18]. These functional issues are especially important in the management of foot and ankle STS,[19–20] since below-knee amputation and prosthetic fitting provides excellent function and versatility in daily activities as well as the capacity to engage in recreational activities.[21,22]

The functional impact of limb-salvage surgery versus amputation was first addressed by Sugarbaker.[20] Despite a variety of methodological problems with that study, it was apparent that both oncological and functional outcome need to be addressed to evaluate limb preservation in the management of soft tissue tumours. The purpose of this study is to describe the management strategy that we employed in a group of patients with localized STS or fibromatosis in the foot and ankle, and to emphasize the functional status of the patients who underwent limb salvage.

### Subjects and methods

From 1987 to 1994, 36 consecutive cases meeting the following inclusion criteria were managed by a multi-disciplinary sarcoma group:

(1) histologically confirmed STS or fibromatosis prior to definitive management;
(2) location of the lesion in the distal one-third of the tibia or the foot such that ablative surgery would require a below-knee amputation;
(3) no metastatic disease on pre-operative systemic staging;
(4) minimum prospective follow-up of 24 months.

Patients with both *de novo* and locally recurrent disease were included.

Initial patient work-up included chest radiography, chest computed tomography (CT), technetium bone scan when indicated, plain films of the distal lower extremity, and axial imaging of the lesion with either CT or magnetic resonance imaging (MRI). Local staging of the extent of the disease identified patients eligible for limb-salvage surgery, while cases that were deemed unresectable were offered below-knee amputation. Criteria used to assess resectability included clinical and radiological assessment of tumour extent and invasion of vital structures, previous surgical incisions and prior management. All pathological diagnosis and assessment of surgical margins were performed by experienced musculoskeletal oncological pathologists. The surgical margin was assessed using both the operative note and pathology report. Histological evidence of disease at the inked surface of the specimen was considered to be a positive margin.[2,3] The decision for adjuvant chemotherapy or radiotherapy was made by the multi-disciplinary team.

Adjuvant radiotherapy was administered using either pre-operative or post-operative protocols. Pre-operative treatment entailed 50 Gy in 25 fractions over 5 weeks. In the early period of the study, most patients received a post-operative boost of 16 Gy in eight fractions commencing after wound healing. However, in the later stages the boost was restricted to those cases in which the margins of the resected specimen were microscopically positive at the time of surgery. A full course of post-operative radiotherapy consisted of 66 Gy in 33 fractions commencing after wound healing. This technique has been described in previous publications.[5,23]

Of the 36 cases (in 35 patients) presenting with STS or fibromatosis in the distal one-third of the leg or foot, four were unresectable and underwent below-knee amputation. The remaining 32 cases were initially deemed appropriate for limb-salvage surgery, but two patients were found at the time of surgery to be unresectable with adequate margins. Following further discussion, these two patients were treated with below-knee amputation.

Data collected included: patient demographics and symptoms; work status; pre-operative functional status; neurovascular involvement; and tumour location, size, histological type and grade. Sarcoma

**Table 1.** *The histological types of ankle and foot tumours. Thirty cases underwent limb salvage, and six cases had below-knee amputation (shown in brackets)*

| | Soft tissue sarcoma | | |
|---|---|---|---|
| Histological type | High grade | Low grade | Fibromatosis |
| Malignant fibrous histiocytoma | 7 (1) | — | — |
| Fibrosarcoma | 2 | 2 | — |
| Leiomyosarcoma | 2 (1) | — | — |
| Clear cell sarcoma | 2 (2) | — | — |
| Synovial cell sarcoma | 1 (1) | 1 | — |
| Liposarcoma | 1 | 1 | — |
| Soft tissue chondrosarcoma | — | 1 (1) | — |
| Neurosarcoma | 1 | — | — |
| Epitheliod sarcoma | 1 | — | — |
| Rhabdomyosarcoma | 1 | — | — |
| STS—unclassified | 1 | — | — |
| Aggressive fibromatosis | — | — | 6 |
| Total 30 + (6) = 36 | 19 (5) | 5 (1) | 6 |

histological grade was designated as low- or high-grade malignancy[24] to allow consistency for comparison between other studies.[2,3,7]. Chemotherapy and radiotherapy treatment and surgical procedures and reconstruction characteristics were recorded. Oncologic outcome with respect to local and systemic recurrence, as well as survival, were documented.

The 30 cases (in 29 patients) treated with limb preservation were followed prospectively and functional outcome was documented by a single physiotherapist at 3, 6, 12 months post-operatively, and at most recent visit to evaluate symptoms, work status, oedema,[25] use of walking aids and the parameters necessary to score the modified Musculoskeletal Tumour Society (MSTS) functional rating scale.[26]. The maximum score is 35 and the seven items included are: pain, range of motion, strength, stability, deformity, functional activity and emotional acceptance of their treatment. Work demands were classified into three groups:

(1) a job requiring heavy physical activity was defined as requiring the majority of time standing or walking with performance of strenuous activities, e.g. lifting, climbing;
(2) domestic work that also required spending a large proportion of time standing but did not require strenuous physical activities;
(3) a non-physical occupation encompassed jobs requiring less than 50% total time standing or walking, e.g. desk work.

Due to the small number of patients, descriptive analysis was used for demographic and tumour-related variables. Repeated measures analysis of variance was used to analyze functional status as measured by the MSTS.

### Results

### *Limb-salvage group*

*Patient characteristics.* Twenty-nine patients underwent limb-salvage surgery, with one patient having bilateral extensive fibromatosis and requiring surgery on each foot. Sixteen patients were females and 13 patients were males, with a mean age of 51 years (range 15–78, SD = 18.5). Mean follow-up was 52 months (range 24–109, SD = 23.2).

At the time of presentation, 20 patients (67%) had undergone either an excisional biopsy or had a local recurrence. Only three patients (10%) were *de novo* presentations, of which two were fibromatosis and the remaining seven were seen following incisional biopsy performed elsewhere. In the majority of patients referred following an excisional biopsy the diagnosis of STS was unsuspected at the time of initial surgery.

Two patients were treated with chemotherapy prior to referral to the multi-disciplinary sarcoma group. One patient had an embryonal rhabdomyosarcoma with metastatic disease at presentation and underwent thoracotomy following chemotherapy. She was free of systemic disease 2 years later and underwent limb salvage with curative intent. She remains alive with no evidence of disease (ANED) 85 months after limb salvage. The second patient received one course of chemotherapy prior to referral but locally progressed.

*Tumour characteristics.* There were 19 high-grade sarcomas, five low-grade sarcomas and six fibromatoses. Histological types are shown in Table 1, with malignant fibrous histiocytoma (MFH) most frequent in our series (23%), followed by fibrosarcoma in four patients (13%). Synovial sarcoma was seen in only one of the limb-salvage patients. The mean tumour size was 6.4 cm and 17 of the lesions (57%) were greater than 5 cm in maximum diameter. Four lesions were larger than 10 cm comprising two fibromatoses, an MFH and a sclerosing liposarcoma.

Thirteen of the lesions were situated around the ankle, nine tumours were located on the dorsum of the foot and seven lesions were on the plantar aspect of the foot; one sarcoma involved the great toe. Four of the fibromatoses were on the plantar aspect of the foot with the remaining two involving the lateral ankle. Lesions were classified as sub-fascial if they were initially subcutaneous but at time of presentation had gross sub-fascial disease or definite evidence of sub-fascial extension from previous surgery. Twenty-eight (93%) of the lesions were sub-fascial at time of presentation and seventeen were invasive, with direct involvement of bone (present in 10 cases), nerve or vessel (present in nine cases).

*Limb-salvage procedure and adjuvant radiotherapy.* Limb salvage was performed by *en bloc* surgery with removal of a cuff of normal tissue around the lesion in 29 cases. In one case of fibromatosis, an intralesional resection was performed. The mean resection specimen size was 11.3 cm and bone resection was required in 10 patients. Reconstruction with a structural bone graft was carried out in three patients. Dorsalis pedis or posterior tibialis vessel resection was performed in seven patients and the superficial peroneal nerve was sacrificed in four. Free muscle flap reconstruction utilized the rectus in five patients, the latissimus dorsi in two patients and the gracilis in one. Split thickness skin graft (STSG) alone was used in seven patients.

Twenty-five patients received adjuvant radiotherapy consisting of pre-operative treatment in eight patients, pre-operative plus post-operative boost in five cases, and post-operative treatment only in 12 cases. In the eight patients treated with free flap reconstructions, adjuvant radiotherapy was used pre-operatively in two patients, pre-operatively plus post-operative boost in three patients, and post-operatively only in three. Twenty-six patients had negative microscopic margins, of whom 21 patients received adjuvant radiotherapy. Each of the three patients with microscopically positive resection margins and the one patient with fibromatosis treated by intralesional surgery received adjuvant radiotherapy.

*Post-operative complications.* Post-operative complications comprised four major wound problems, two minor wound infections and one pulmonary embolus. The four major wound infections required debridement and healed by secondary intention. One of these was in a patient with a foot lesion who received post-operative radiotherapy to an STSG which became secondarily infected.

Five patients suffered fractures within the high-dose irradiation field 4–19 months following surgery. Three of these patients had required partial bone resection. There were two patients requiring partial resections of the anterior tibia with ankle arthrodesis, one with resection of the anterior talus, and one patient who underwent partial resection of the fibula *en bloc* with the tumour. The fifth patient with a fracture had a drill hole passed through the first metatarsal as part of her reconstructive procedure. The fractures healed in all but one patient who suffered a fracture through the anterior tibial bony defect. This patient went on to chronic non-union despite bone grafting and ultimately required below-knee amputation.

*Functional outcome.* Complete functional results were available on 27 of the 30 consecutive cases (90%) that underwent limb-salvage surgery (Table 2). The remaining three patients do not live close to our centre and are followed near their home. Measurements of the MSTS functional rating scale at 3, 6, 12 months and most recent follow-up show a gradual improvement in the functional score up to 12 months and a plateau beyond one year. This finding was not statistically significant (overall $F$-test, $p = 0.19$). Fifteen patients were rated excellent, eight good, and four fair or poor, at most recent follow-up. Only two patients showed a deterioration of limb function over time. One patient treated by surgery and irradiation to 66 Gy developed recreational restrictions and moderate pain one year following treatment. The second patient required amputation for complications of limb-salvage. At the most recent visit, all but four patients were enthusiastic about their limb-salvage result, except four who reported being satisfied with the result.

Six patients were unable to return to their premorbid employment. Three of six patients previously employed as labourers were unable to return to work. This contrasts with the less physically-demanding occupations in which 88% were able to return to their premorbid level of activity. No patients remaining off work at one year after surgery were able to return to their original employment. After one year, no patient had an improvement in their overall functional rating, or improvement in the clinical measurements of the MSTS functional evaluation (motion, stability, deformity and strength). Two patients, however, reported an improvement in their functional activity at most recent follow-up which may suggest an adaptation to physical disability. Only three patients required an ambulatory aid at most recent assessment compared to nine patients at 3 months after surgery. Two patients continue to use an orthosis, and two use a rocker bottom sole on their footwear.

At 3 months after limb-salvage surgery, 56% of patients had some degree of lower limb oedema. At most recent assessment, six patients (22%)

**Table 2.** *Functional status at 3, 6, 12 months, and most recent follow-up* (n = 27, *missing* = 3)

| Status | 3 months | 6 months | 12 months | Most recent |
|---|---|---|---|---|
| MSTS | | | | |
| Mean | 27.45 | 29.93 | 31.42 | 31.00 |
| SD | 8.16 | 6.82 | 4.64 | 5.69 |
| Range | 11–35 | 13–35 | 17–35 | 13–35 |
| Work status* | | | | |
| Labourer | 0/6 | 1/6 | 2/6 | 3/6 |
| Domestic | 4/5 | 4/5 | 4/5 | 4/5 |
| Non-physical | 13/16 | 14/16 | 14/16 | 14/16 |
| Requiring gait aid | | | | |
| None | 18 | 21 | 22 | 24 |
| Cane | 5 | 3 | 4 | 2 |
| Crutches | 3 | 3 | 1 | 1 |
| Walker | 1 | 0 | 0 | 0 |
| Persisting oedema** | | | | |
| None | 12 | 16 | 16 | 21 |
| Mild | 8 | 8 | 9 | 5 |
| Moderate | 6 | 2 | 2 | 1 |
| Severe | 1 | 1 | 0 | 0 |

*Able to return to premorbid work.
**Criteria as per Stern.[25]

continued to have mild leg oedema (Table 2). Thirteen patients reported persistent pain, with the majority having mild pain without need for narcotic agents but two patients continued to require occasional narcotic use for severe pain.

*Oncologic outcome.* At mean follow-up of 52 months (24–109 months), there were four systemic recurrences in patients with high-grade tumours, one local recurrence in a patient with fibromatosis and one regional recurrence in a second patient with fibromatosis (Table 3). The single patient experiencing local recurrence had undergone limb salvage for a small (<5 cm), *de novo* symptomatic fibromatosis on the plantar aspect of the foot. The lesion was resected with wide margins and no adjuvant radiotherapy was used; recurrent fibromatosis developed 14 months after treatment. This patient has had no further intervention and clinically is stable with a good functional result. The patient who developed regional recurrence had undergone below-knee amputation for complications of limb salvage and subsequently developed recurrence in her stump. This recurrence was far removed from the original lesion and more than 10 cm outside the radiation field used 3 years before. She is currently disease free following above-knee amputation and functioning with a prosthesis. Three patients are dead of disease and one patient is alive with pulmonary metastasis. All four patients had high-grade lesions and developed systemic recurrence less than one year following surgery (mean 7 months). Three of the lesions showed invasive properties, either encasing neurovascular structures or eroding into adjacent bone. In total, 24 (80%) patients remain disease free and 25 patients are currently alive with no evidence of disease.

*Amputation group*

Six patients (four males and two females) with a mean age of 52 years had below-knee amputations for local disease; they had no metastatic disease on pre-operative staging. Four patients were preoperatively considered unresectable due to the extensive involvement of local vital structures, while the other two were found at the time of surgery to be unresectable and underwent amputation shortly thereafter. This group consisted of five high-grade lesions and one low-grade STS (Table 1). Two sarcomas presented *de novo*, one following incisional biopsy, and three after attempted excisional biopsy and tumour contamination throughout the foot. Three lesions originated around the ankle, two lesions involved the dorsal foot and one was situated on the plantar aspect of the foot. With a mean follow-up time of 26 months for the amputated patients, three continue to be disease free. Two patients are dead of disease and a third is alive with systemic disease.

**Table 3.** *Oncologic outcome in limb-salvage cases* (n = 30) *and patients with below-knee amputation* (n = 6)

| Outcome | Limb salvage (%) | Amputation (%) |
|---|---|---|
| Dead of disease | 3 (10) | 2 (33) |
| Alive, with evidence of disease | 1 | 1 |
| Local recurrence | 1 | — |
| Regional recurrence | 1* | — |
| Disease-free survival | 24 (80) | 3 (50)** |

*One patient developed recurrent fibromatosis proximal to the stump, following below-knee amputation for complication of limb salvage.

**One patient lost to follow-up 14 months post-amputation.

### Discussion

STS sarcoma of the foot and ankle poses a difficult challenge for limb preservation. With the success of combined modality therapy in proximal extremity STS, similar principles are being applied to achieve limb salvage of the distal lower limb.[7,9,13,18,27] This paper presents 36 consecutive cases of STS or aggressive fibromatosis treated by multi-modality therapy by our sarcoma group. Patients with aggressive fibromatosis requiring resection were included in the study since the treatment of this disease often requires combined management with both surgery and adjuvant radiotherapy. Since the therapeutic approach to both STS and fibromatosis was similar in our hands, we grouped these patients together in order to gain a better understanding of the functional results of treatment. Thirty cases underwent limb salvage (83%) employing a consistently applied protocol of wide local *en bloc* resection plus adjuvant radiotherapy to supplement close surgical margins. Since the ultimate goal in these patients is overall survival and preservation of maximum limb function, a concurrent analysis of both outcomes is appropriate.

Sugarbaker *et al.*,[20] in a landmark paper on quality of life and functional assessment, did not substantiate an improved function in limb salvage versus amputation for STS of the upper and lower extremity. Patients were randomized to amputation or limb salvage, with quality of life assessment at one point in time. In their paper, Sugarbaker *et al.* questioned the results of the study due to the generalizability and sensitivity of the outcomes administered. Currently, when possible, limb preservation is preferred over amputation, even in the absence of sound evidence in the literature.[7,9,13,18,27,28] This study provides objective results on the functional benefit of limb-salvage surgery in patients with localized soft tissue tumours of the foot and ankle.

While the MSTS[26] does not meet the current standards of evaluating function from the patient's perspective,[29] we chose to evaluate function for this study using the MSTS, 1987 version, as it was the only measure available specific to the tumour limb-salvage population at the time that prospective functional data collection was begun. While it is recognized that the MSTS includes many items related to clinical parameters, e.g. range of motion, strength, joint stability and joint deformity, this scale does provide data that can be compared to other published studies. Furthermore, we have added an evaluation of work status in relation to job demands as an indicator of function.

Eighty-five per cent of the patients had a good or excellent functional outcome at most recent visit. Sixteen patients had no functional limitations and all patients were satisfied with the result of their treatment. The only exception was the patient who required amputation for complication of treatment. Fifty-two per cent had no pain and only two patients required occasional narcotic use. The MSTS functional evaluation rating scale showed a progressive improvement in function with time and a plateau of function after one year. This correlates with the finding that patients returned to work up to one year following treatment but not beyond that time. This information has implications with respect to patient and physician expectations over time and the planning of post-operative physiotherapy.

Chou and Malawer[13] reviewed 33 patients with an assortment of bone or soft tissue lesions of the foot and ankle. Surgical management varied from simple curettage to amputation. They reported that 82% of patients had a good to excellent functional outcome according to the MSTS functional evaluation criteria and 55% were able to bear full weight and had unlimited activity.[14] Supporting documentation was not provided and it is not certain whether any patients received radiotherapy. Nonetheless, their population was confined to the foot and ankle and provides one of the few papers in which a comparison of the functional outcome can be made with our study.

Owens *et al.*, in a retrospective study of 50 patients with stage $M_0$ STS of the hand and foot, emphasized that while local recurrence had an ominous effect on prognosis, an improvement in local control did not translate into an improved survival. Approximately half of their patients had amputation and the remainder had various combinations of combined modality limb salvage.[7] A 5-year survival of 68% with either amputation (no local recurrence) or conservative surgery (32% local recurrence) was reported, a finding shared by other authors.[2–4,7,17,28] In our group of limb-salvage patients, no sarcoma recurred locally although there was one local and one regional recurrence of fibromatosis (7%). There was a 20% systemic recurrence rate at a mean time of 7 months from presentation in patients with STS. Heise *et al.*, in an extensive review of extremity STS, showed a median time to recurrence of 9.7 months for local and systemic recurrence combined.[30] The presence of occult metastatic disease at time of presentation continues to be a challenge, especially since no therapeutic interventions have thus far been shown to improve prognosis.[31]

Talbert *et al.* reported on 78 patients with conservative surgery and irradiation of the hand and foot.[18] Nine patients had definite residual gross disease and no further surgery, while 46 patients with piecemeal or simple local excision had no further surgery. All patients were treated with a combination of adjuvant radiotherapy and chemotherapy. There was a 19% local recurrence rate and the complication rate was 56% in the lower extremity versus 28% in the upper extremity. Thirteen per cent of the lower extremity cases required amputation for complication of their treatment. Fifty-three per cent of the distal lower extremity patients had normal function or mild to moderate functional limitation. The use of radiotherapy to control local disease when there is histological or gross evidence of residual disease may not be prudent and the larger dose required may lead to increased functional morbidity.[2,5,7,8,31]

Stinson *et al.* described both the frequent long-term functional complications of radiotherapy and methods to reduce their incidence.[33] They reported a third of their patients had a moderate to severe decrease in range of motion and a 19% rate of oedema greater than 2+. Twenty-two per cent of our patients had mild (1+) or moderate (2+) oedema using the criteria of Stern[25] at last visit. Except in two patients, delayed local toxic effects of adjuvant radiotherapy did not translate into a deterioration of function with time as shown in other studies.[18,32,34] One patient had increasing pain in the foot one year following radiotherapy. The second patient required amputation for pathological fracture and non-union 2 years after limb salvage. Improvement in radiotherapy technique has enabled more accurate dosage to specific anatomical regions, the sparing of part of the limb circumference and the use of a more standardized, better tolerated dose, reducing the risk of complications following irradiation.[5,23]

The combination of poor soft tissue coverage following resection in a distal extremity that also receives adjuvant radiotherapy can represent a major challenge to the reconstructive surgeon. The use of vascularized tissue transfer from distant sites brings both soft tissue coverage and new blood supply to the area. A review of wound healing complications after extremity STS surgery and adjuvant radiotherapy by Peat *et al.* showed an 11% major wound complication rate with the use of vascularized tissue transfers versus a 30% rate of complications with direct wound closure.[23] In our series, two of eight tissue transfers had major problems but only one was not salvageable. Six of the vascularized flaps received post-operative radiotherapy of which one developed partial flap necrosis. The residual flap survived but the patient ultimately required amputation for a pathological fracture and non-union. The successful use of a vascularized tissue transfer in seven out of eight otherwise unreconstructable limb-salvage patients supports the benefit of this technique in limb preservation surgery of the foot and ankle.

A pathological fracture in the high-dose irradiation field (as experienced by five patients in this series) presents potentially devastating consequences for limb function as demonstrated by the one patient who required below-knee amputation for chronic non-union. The two patients with the anterior tibia cortical defects were part of a series of patients in whom it was demonstrated that open segmental cortical defects with adjuvant irradiation increase the risk for pathological fracture.[3,5] Our current practice when treating such patients is to stabilize prophylatically the weakened bone with an intramedullary rod, plate or bone graft. These fractures may have been prevented using our current management protocol.

Most published studies reporting outcome in foot and ankle sarcoma have concentrated on oncologic outcome with only a minor emphasis on functional issues. The modified MSTS functional evaluation rating scale provides a means of assessing function in the extremity. Combined with the excellent local control achieved in this study the functional results provide support for limb salvage as a primary goal in the management of selected patients with STS of the foot and ankle. Concurrent measurement of function and oncologic outcome will better enable us to assess future treatment interventions in the management of STS of the extremities.

### References

1 Simon MA, Enneking WF. The management of soft-tissue sarcomas of the extremities. *J Bone Joint Surg* 1976; 58A:317–27.
2 Bell RS, O'Sullivan B, Liu FF, *et al.* The surgical margin in soft-tissue sarcoma. *J Bone Joint Surg* 1989; 71A:370–5.
3 Brennan MF, Casper ES, Harrison LB, *et al.* The role of multimodality therapy in soft-tissue sarcoma. *Ann Surg* 1991; 214:328–36.
4 Brennan MF, Hilaris B, Shiu MH, *et al.* Local recurrence in adult soft-tissue sarcoma. *Arch Surg* 1987; 122:1289–93.
5 LeVay J, O'Sullivan B, Catton C, *et al.* Outcome and prognostic factors in soft tissue sarcoma in the adult. *Int J Radiat Oncol Biol Phys* 1993; 27:1091–9.
6 O'Connor MI, Pritchard DJ, Gunderson LL. Integration of limb-sparing surgery, brachytherapy, and external-beam irradiation in the treatment of soft-tissue sarcomas. *Clin Orthop* 1993; 289:73–80.
7 Owens JC, Shiu MH, Smith R, *et al.* Soft tissue sarcomas of the hand and foot. *Cancer* 1985; 55:2010–18.
8 Rosenberg SA, Tepper J, Glatstein E, *et al.* The treatment of soft-tissue sarcomas of the extremities. *Ann Surg* 1982; 196:305–14.
9 Johstone PA, Wexler LH, Venzon DJ, *et al.* Sarcomas of the hand and foot; analysis of local control and functional result with combined modality therapy in extremity preservation. *Int J Radiat Oncol Biol Phys* 1994; 29:735–45.
10 Karakousis CP, Mayordomo J, Zografos GC, *et al.* Desmoid tumors of the trunk and extremity. *Cancer* 1993; 72:1637–41.
11 McCollough WM, Parsons JT, Van Der Griend R, *et al.* Radiation therapy for aggressive fibromatosis. *J Bone Joint Surg* 1991; 73A:717–25.
12 Suit HD. Radiation dose and response of desmoid tumors. *Int J Radiat Oncol Biol Phys* 1990; 19:225–7.
13 Chou LB, Malawer MM. Analysis of surgical treatment of 33 foot and ankle tumors. *Foot Ankle Int* 1994; 15:175–81.
14 Kirby EJ, Shereff MJ, Lewis MM. Soft-tissue tumors and tumor-like lesions of the foot. *J Bone Joint Surg* 1989; 71A:621–9.
15 Seale KS, Lange TA, Monson D, *et al.* Soft tissue tumors of the foot and ankle. *Foot and Ankle* 1988; 9:19–27.

16 Harrison LB, Franzese F, Gaynor JJ, *et al.* Long-term results of a prospective randomized trial of adjuvant brachytherapy in the management of completely resected soft tissue sarcomas of the extremity and superficial trunk. *Int J Radiat Oncol Biol Phys* 1993; 27:259–65.
17 Suit HD, Mankin HJ, Wood WC, *et al.* Treatment of the patient with stage Mo soft tissue sarcoma. *J Clin Oncol* 1988; 6:854–62.
18 Talbert ML, Zagars GK, Sherman NE, *et al.* Conservation surgery and radiation therapy for soft tissue sarcoma of the wrist, hand, ankle, and foot. *Cancer* 1990; 66:2482–91.
19 Kaasa S. Measurement of quality of life in clinical trials. *Oncology* 1992; 49:288–94.
20 Sugarbaker PH, Barofsky I, Rosenberg SA, *et al.* Quality of life assessment of patients in extremity sarcoma clinical trials. *Surgery* 1982; 91:17–23.
21 Enoka RM, Miller DI, Burgess EM. Below-knee amputee running gait. *Am J Phys Med* 1982; 61:66–84.
22 Michael JW, Gailey RS, Bowker JF. New developments in recreational prostheses and adaptive devices for the amputee. *Clin Orthop* 1990; 256:64–75.
23 Peat BG, Bell RS, Davis AM, *et al.* Wound-healing complications after soft-tissue sarcoma surgery. *Plast Reconstr Surg* 1994; 93:980–7.
24 Enneking WF, Spanier SS, Goodman MA. A system for the surgical staging of musculoskeletal sarcoma. *Clin Orthop* 1980; 153:106–20.
25 Stern TN. *Clinical examination: a textbook of physical diagnosis*. Chicago: Year Book Medical Publishers, 1964.
26 Enneking WF. Modified system for functional evaluation of surgical management of musculoskeletal tumors. In: Enneking WF, ed. *Limb salvage in musculoskeletal oncology*. New York: Churchill Livingstone, 1987; 626–37.
27 Kinsella TJ, Loeffler JS, Fraass BA, *et al.* Extremity preservation by combined modality therapy in sarcomas of the hand and foot: an analysis of local control, disease free survival and functional result. *Int J Radiat Oncol Biol Phys* 1983; 9:1115–19.
28 Gwin LJ, Bell JL. Optimizing local control in soft tissue sarcoma of the extremity. *Oncology* 1994; 8:25–41.
29 Geigle R, Jones SB. Outcome measurement: a report from the front. *Inquiry*, 1990; 27:7–13.
30 Heise WH, Myers MH, Russell WO, *et al.* Recurrence-free survival for surgically treated soft tissue sarcoma patients: multivariate analysis of five prognostic actors. *Cancer* 1986; 57:172–7.
31 Bramwell V, Rouesse J, Steward W, *et al.* Adjuvant CYVADIC chemotherapy for adult soft tissue sarcoma—reduced local recurrence but no improvement in survival: a study of the European Organization for Research and Treatment of Cancer soft tissue and bone sarcoma group. *J Clin Oncol* 1994; 12:1137–49.
32 Karasek K, Constine LS, Rosier R. Sarcoma therapy: functional outcome and relationship to treatment parameters. *Int J Radiat Oncol Biol Phys* 1992; 24:651–6.
33 Stinson SF, DeLaney TF, Greenberg J, *et al.* Acute and long-term effects on limb function of combined modality limb sparing therapy for extremity soft tissue sarcoma. *Int J Radiat Oncol Biol Phys* 1991; 21:1493–9.
34 Suit HD, Russell WO, Martin RG. Management of patients with sarcoma of soft tissue in the extremity. *Cancer* 1973; 31:1247–55.
35 Bell RS, O'Sullivan B, Nguyen C, *et al.* Fractures following limb-salvage surgery and adjuvant irradiation for soft-tissue sarcoma. *Clin Orthop* 1991; 271:265–71.
